# Genome Sequence of the Red Pigment-Forming *Meiothermus taiwanensis* Strain RP Isolated from Paniphala Hot Spring, India

**DOI:** 10.1128/genomeA.00629-16

**Published:** 2016-06-30

**Authors:** Trinetra Mukherjee, Sucharita Bose, Urmimala Sen, Chayan Roy, Moidu Jameela Rameez, Wriddhiman Ghosh, Subhra Kanti Mukhopadhyay

**Affiliations:** a Department of Microbiology, The University of Burdwan, Burdwan, West Bengal, India; b Bose Institute, Centenary Campus, Department of Microbiology, Kolkata, West Bengal, India

## Abstract

Here we report the draft genome sequence of *Meiothermus taiwanensis* strain RP (MCC 2966), isolated from the Paniphala hot spring of India, which contains genes encoding for enzymes of the methyl erythritol 4-phosphate (MEP) pathway of isoprenoid biosynthesis and carotenoid backbone synthesis.

## GENOME ANNOUNCEMENT

*Meiothermus taiwanensis* strain RP (MCC 2966) is a thermophilic, heterotrophic, aerobic bacterium belonging to the family *Thermaceae* of the phylum *Deinococcus-Thermus*. The strain was isolated from a water sample from the Panifala hot spring near Barabani (23°45′33″N, 86°58′54″E) in the Burdwan district, West Bengal, India, plated onto R2A agar medium and incubated at 60 ± 1°C for 24 to 48 h, where it formed red-pigmented colonies.

Purified genomic DNA from the isolate was subjected to genome sequencing on an Ion PGM sequencer (1) using a 318 chip. 517,140 raw reads were assembled by MIRA version 4.9.5.2 into 210 contigs at 40.82 % coverage. An estimated 2,988,016-bp genome was obtained, the largest contig size being 114,376 bp and the smallest one being 205 bp with an average G+C content of 64%. The genome was annotated using results from the RAST server (2), Patric (3), and manual analysis leading to prediction of ~3,029 protein coding sequences compared to 2,964 protein coding genes predicted in the type strain *Meiothermus taiwanensis* strain DSM 14542 genome.

Among the two pathways for isoprenoid backbone biosynthesis, the genome assembly data suggest that the MEP (methyl erythritol 4-phosphate) pathway is utililized through the gene products 1-deoxy-d-xylulose-5-phosphate synthase, 1-deoxy-d-xylulose-5-phosphate reductoisomerase, 3-ketoacyl Co-A thiolase, dimethylallyl transferase, 1-hydroxyl-2-methyl-2-(E)-butenyl 4-diphosphate synthase, isopentenyl diphosphate delta-isomerase, geranylgeranyl diphosphate synthase, etc., which lead to formation of geranylgeranyl pyrophosphate. Genes have been found encoding for five key enzymes of carotenoid backbone synthesis, phytoene synthase, phytoene desaturase (neurosporene or lycopene producing), beta-carotene ketolase, lycopene beta cyclase, and phytoene dehydrogenase leading to canthaxanthin formation (4). Previously, species of *Meiothermus* have been found to produce red or yellow colored pigments and some of them were identified from *M. ruber* as 1′-β-glucopyranosyl-3,4,3′,4′-tetradehydro-1′,2′-dihydro-β,ψ-caroten-2-one glucose acetylated at position 6 along with a series of carotenoid glycoside esters. The data from the whole genome suggest the formation of the β-carotene backbone whose modifications probably lead to the formation of other carotenoids related to previously found carotenoids of *M. ruber* (5, 6).

Multiple genes related to resistance toward copper, cobalt, cadmium, chromium, etc., have been found along with genes related to the *Mycobacterium* virulence operon and *Listeria* surface protein. Enzymes of the central metabolic pathway (glycolysis, tricarboxylic acid [TCA] cycle, pentose phosphate pathway, glyoxylate cycle, and oxidative phosphorylation) indicate an aerobic mode of respiration and a heterotrophic mode of carbon utilization. Some fermentative pathway genes for butanol, acetolactate, and butyrate formation are present. Genes found useful for some other functions include auxin and polyhydroxybutyrate metabolism, aromatic dioxygenase, synthesis of capsular polysaccharide components sialic acid and rhamnose, uptake and biosynthesis of compatible solutes, ammonia assimilation, inorganic sulfur assimilation, and sulfur oxidation.

### Nucleotide sequence accession numbers.

This whole-genome shotgun project has been deposited at DDBJ/ENA/GenBank under the accession LWCO00000000. The version described in this paper is version LWCO01000000.
